# Plant DNA Polymerases

**DOI:** 10.3390/ijms20194814

**Published:** 2019-09-27

**Authors:** Jose-Antonio Pedroza-Garcia, Lieven De Veylder, Cécile Raynaud

**Affiliations:** 1 Department of Plant Biotechnology and Bioinformatics, Ghent University, B-9052 Ghent, Belgium; livey@psb.vib-ugent.be; 2 VIB Center for Plant Systems Biology, B-9052 Ghent, Belgium; 3 Institute of Plant Sciences Paris-Saclay (IPS2), CNRS, Bâtiment 630, 91405 Orsay, France; cecile.raynaud@ips2.universite-paris-saclay.fr; 4 Institute of Plant Sciences Paris-Saclay (IPS2), INRA, Bâtiment 630, 91405 Orsay, France; 5 Institute of Plant Sciences Paris-Saclay (IPS2), Paris-Sud University, Bâtiment 630, 91405 Orsay, France; 6 Institute of Plant Sciences Paris-Saclay (IPS2), Univeristy of Evry, Bâtiment 630, 91405 Orsay, France; 7 Institute of Plant Sciences Paris-Saclay (IPS2), Paris-Diderot University, Batiment 630, 91405 Orsay, France; 8 Institute of Plant Sciences Paris-Saclay (IPS2), Sorbonne Paris-Cité, Bâtiment 630, 91405 Orsay, France; 9 Institute of Plant Sciences Paris-Saclay (IPS2), Paris-Saclay Univeristy, Bâtiment 630, 91405 Orsay, France

**Keywords:** DNA replication, DNA repair

## Abstract

Maintenance of genome integrity is a key process in all organisms. DNA polymerases (Pols) are central players in this process as they are in charge of the faithful reproduction of the genetic information, as well as of DNA repair. Interestingly, all eukaryotes possess a large repertoire of polymerases. Three protein complexes, DNA Pol α, δ, and ε, are in charge of nuclear DNA replication. These enzymes have the fidelity and processivity required to replicate long DNA sequences, but DNA lesions can block their progression. Consequently, eukaryotic genomes also encode a variable number of specialized polymerases (between five and 16 depending on the organism) that are involved in the replication of damaged DNA, DNA repair, and organellar DNA replication. This diversity of enzymes likely stems from their ability to bypass specific types of lesions. In the past 10–15 years, our knowledge regarding plant DNA polymerases dramatically increased. In this review, we discuss these recent findings and compare acquired knowledge in plants to data obtained in other eukaryotes. We also discuss the emerging links between genome and epigenome replication.

## 1. Introduction

Maintenance of genome integrity is crucial to achieve faithful transmission of the genetic information in proliferating cells and from one generation to the next. DNA polymerase (Pol) enzymes play a key role in this process because they perform DNA synthesis during the replication phase of the cell cycle and DNA repair.

The genome of each organism encodes several DNA polymerases. To date, five DNA polymerases were characterized in *Escherichia coli*, eight in *Saccharomyces cerevisae*, and as many as 16 in human [1]. In *Arabidopsis thaliana*, 10 orthologues of human polymerases were identified, plus two additional polymerases that are involved in the replication and repair of the organellar DNA [2]. DNA polymerases are generally classified into different families (A, B, X, and Y) depending of the primary structure of their catalytic subunit [3]; non-replicative polymerases involved in translesion synthesis (TLS, a process that allows DNA replication to proceed passed DNA lesions), organelle DNA metabolism, or nuclear DNA repair are found in all families, whereas eukaryotic replicative polymerases all belong to the B family. Replicative and TLS DNA polymerases can differ broadly in terms of error rate and processivity. The latter is defined as the number of bases added in a single contact event, since DNA polymerases constantly associate to and dissociate from their template. Replicative DNA polymerases that need to synthesize large amounts of DNA within a short period of time and generate as few errors as possible typically show the lowest error rate and highest processivity, whereas TLS polymerases have a more open active site that allows them to accommodate lesions, resulting in a higher error rate [4,5]. In this review, we do not discuss the complex structural properties of DNA Pol families that were recently reviewed elsewhere [1].

In humans, mutations affecting DNA Pols contribute to various disorders, including cancer and/or developmental defects [6,7]. Although carcinogenesis does not occur in plants, inadequate DNA replication and repair lead to growth inhibition and developmental defects. Therefore, exposure to various types of stresses that can compromise DNA replication or induce DNA damage can reduce the plant’s fitness. The last review about plant DNA polymerases was published 12 years ago by Garcia-Diaz and Bebenek [2], when the experimental evidence was scarce in the field. During the last decade, the roles of plant DNA polymerases were investigated into more detail, shedding light on their conserved and unique roles. Here, we describe our current understanding on plant DNA polymerases, discussing both common features with their homologues in animals or yeast and unique specificities to highlight the questions that remain open in the field.

## 2. Replicative DNA Polymerases, Guardians of the Genome and Epigenome Integrity

As mentioned in the introduction, despite the availability of numerous DNA polymerases, only three of them are responsible for genome duplication. Pol δ and Pol ε are the main eukaryotic DNA replicases, and together perform the bulk of DNA replication, following priming by Pol α [8]. These polymerases are actually protein complexes that comprise a large subunit harboring the catalytic activity, and accessory subunits, some of which are dispensable for the DNA synthesis activity. Table 1 shows the formal nomenclature for eukaryotic replicative DNA polymerases in human, yeast (*S. pombe* and *S cerevisiae)*, and *Arabidopsis*.

These three polymerases are members of the B family, characterized by conserved amino-acid motifs within the polymerase catalytic sites and exonuclease domain. Pol δ and ε are characterized by a high fidelity, due to the tight conformation of their active site that allows the incorporation of mismatched nucleotide with a frequency around 10^−7^, as well as to their proof-reading activity that improves fidelity by about 100–1000 [9,10]. Both polymerases associate with the sliding clamp proliferating cell nuclear antigen (PCNA; see below) that enhances their processivity, notably that of Pol δ. By contrast, Pol α, being only responsible for the synthesis of the primers that are then elongated by Pol δ [11], is a DNA polymerase of moderate fidelity that lacks 3′→5′ exonuclease activity [4].

In addition to their role in DNA synthesis, replicative DNA polymerases are involved in the surveillance of stalled forks that may be generated by constraints on the replication machinery such as G-quadruplexes, DNA lesions, or collisions between transcription and replication. In yeast, at least Pol ε and α participate in the activation of the synthesis (S)-phase checkpoint [12,13]. Moreover, replicative DNA polymerases are directly implicated in transferring the epigenetic information to the newly synthesized daughter chromatin strands, thereby maintaining the epigenetic status of the replicated loci [14]; this mechanism allows re-establishing transcriptional silencing once the replication fork passes certain genomic regions [15,16]. In the next section, we describe the subunit composition of plant replicative polymerases and mutant lines that allowed the functional characterization of these proteins, and summarize our current knowledge of these polymerases involvement in DNA replication, DNA repair, and chromatin replication.

## 3. Division of Labor between Replicative Polymerases at the Replication Fork

Replicative polymerases are associated into a large protein complex called the replisome that encompasses all the core activities required for high-fidelity DNA replication [17]. In addition to replicative DNA polymerases, the replisome comprises an 11-subunit helicase complex. The helicase activity is brought by the MCM2–7 (mini chromosome maintenance) heterohexamer that forms a ring unwinding unreplicated DNA. To be activated, the MCM complex needs to associate with the GINS (consisting of four proteins Sld5–Psf1–Psf2–Psf3 also called “go–ichi–ni–san”, from the Japanese for 5–1–2–3) and CDC45. Altogether these subunits form the CMG complex (CDC45, MCM, GINS). Finally, the replisome also comprises the sliding clamp PCNA that is a processivity factor of DNA polymerases δ and ε and the sliding clamp loader RFC (replication factor C), as well as the single-strand DNA binding protein RPA (replication protein A) that coats and protects single-stranded DNA behind the helicase [18]. Replicative Pols play complementary roles during the replication process, and each polymerase, thus, interacts with distinct genetic networks [19]. Firstly, DNA Pol ε is unique in that it is required for the pre-initiation steps of DNA replication, by allowing replisome assembly [20], while Pol α and δ are recruited later on chromatin. Once the replication fork is opened by the CMG, an RNA/DNA primer produced by the DNA Pol α/primase complex initiates leading-strand synthesis and each Okazaki fragment on the lagging strand (reviewed in Reference [21]). Polymerase δ was recently shown to elongate these primers on both strands [22]. Next, the generally accepted view is that Pol δ synthesizes the lagging strand [17], while Pol ε is responsible for the synthesis of the leading strand [23]. Recent work proposed that Pol δ could normally replicates both strands of the DNA, and that, occasionally, a switch to Pol ε on the leading strand could be induced by replication errors, thereby coupling checkpoint signaling to repair of the DNA damage [24]. Such a mechanism would also account for the observation that Pol ε preferentially ensures leading-strand fidelity [25], but it is highly debated [26] and it not supported by other recent studies [22]. Finally, replication termination involves a switch from Pol ε to Pol δ [27]; thus, the commonly accepted model is that Pol δ performs initiation and termination on both strands, as well as the synthesis of Okazaki fragments, whereas Pol ε elongates only the leading strand.

Most of our knowledge on eukaryotic DNA replication was acquired in yeast and animal cells. In plants, homologues of the proteins required for the different steps of replication were identified, especially in *Arabidopsis* and rice [28]. Among the plant replisome proteins that were already studied through genetic approaches are some MCM subunits, CDC45, PCNA, RFC, and RPA [29,30,31,32,33,34]. Likewise, the flap endonuclease and ligase involved in the processing of junctions between Okazaki fragments were identified [35]. The study of protein–protein interactions or biochemical activity of replisome components is still in its infancy but, given the conservation of the essential coding sequences, it is generally assumed that these processes do not differ significantly in plants from what is described in other eukaryotes [36].

In *Arabidopsis*, like in other eukaryotes, knockout mutants of the main subunits of the three replicative DNA polymerase are lethal. However, hypomorphic alleles of catalytic subunits were isolated in genetic screens for mutants deficient for processes as diverse as embryo development, epigenetic silencing, and hormone signaling [37,38,39,40]. This fact illustrates the crucial importance of DNA replication for all aspects of plant development, but also points to the diversity of the functions encompassed by plant replicative DNA polymerases. Like other eukaryotes, DNA Pol α, δ, and ε not only replicate the genome, but also have multiple functions in the maintenance of the genome and epigenome integrity (Table 2).

## 4. Subunit Composition of Plant Replicative Polymerases

### 4.1. DNA Pol ε

In all eukaryotes, DNA Pol ε is a four-subunit complex comprising a catalytic subunit (POLE1/POL2A) and three accessory subunits DPB2, 3, and 4 (DNA PolII subunit B), of which only DPB2 is required for cell viability [70], although it does not seem to be required for the polymerase activity per se [71]. In yeast, the other accessory subunits, DPB3 and 4, are dispensable for cell viability, but their inactivation leads to genetic instability, suggesting that they affect Pol ε fidelity [72]. By contrast, in mouse, the DPB4 subunit is essential for embryo survival because the absence of this subunit destabilizes the whole complex [73].

**Figure 1:**
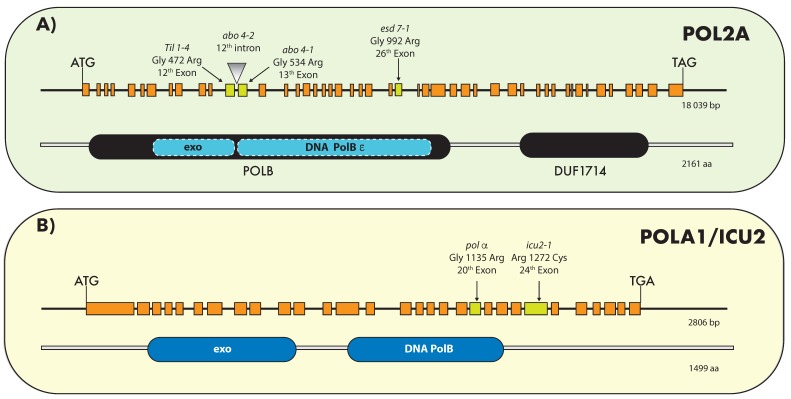

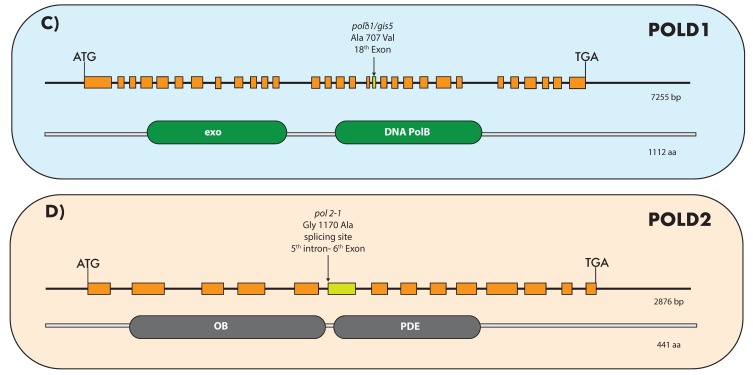
Hyphomorphic alleles of replicative DNA polymerases (Pols) in *Arabidopsis thaliana*. On all panels, orange boxes represent exons, and the black line represents introns and regulatory sequences. Below the gene structure, the schematic organization of the corresponding protein is shown. (**A**) The *AtPOL2A* gene also known as *TIL1/ABO4/ESD7* is annotated to be 18,039 bp, with 48 exons, accounting for an open reading frame of 6818 bp. The *til1-4* plants contain two G-to-A mutations: one at position 3927 (counting from the first ATG) in exon 12 and one at position 5005 in intron 14. The former mutation changes a conserved Gly (position 472) into Arg [37]. The *abo4-1* mutation changes Gly (position 534) to Arg (G to A in position 4171 counting from the first putative ATG, in the 13th exon). The *abo4-2* Salk transfer DNA (T-DNA) insertion line (SALK 0963441) was also found to be viable: in the *abo4-2* mutant, the T-DNA is inserted at position 3972 (in the 12th intron), counting from the first putative ATG of the genomic coding sequence [39]. In the mutant, messenger RNAs (mRNAs) lacking exons 12 and 13 (represented in yellow) are produced [50]. The *esd7-1* mutation consists of a guanine-to-adenine transition in the 26th exon, which substitutes Gly (G) with Arg (R) at amino-acid position 992, a residue located in the catalytic domain [52]. (**B**) Structure of the *POLA1/ICU2* gene and corresponding protein. The *POLA1* gene is 2806 bp long and encodes a 1499-amino-acid (a.a.) protein. The *icu2-1* mutant harbors a point mutation in a C/T transition in the 24th exon, at nucleotide position 6762 from the initiation codon, which substitutes Arg (R) with Cys (C) at amino-acid position 1273 [38]. The *polα* mutation consists of a guanine-to-adenine transition in position 5996 counting from the first putative ATG within the 20th exon, which substitutes Gly (G) with Arg (R) at amino-acid position 1135, a residue in the catalytic domain [41]. (**C**) Structure of the *POLD1* gene and corresponding protein. The *POLD1* gene is 7255 bp long and encodes a protein of 1112 amino acids. The *gis5* mutation is located within the polymerase domain in the 18th exon causing a C-to-T transition which leads to an Ala 707 Val substitution [40]. (**D**) Structure of the *POLD2* gene and corresponding protein. The gene is 2876 bp long and encodes a 441 amino-acid protein. The *pold2-1* mutation changes G to A at position 1170 counting from the first putative ATG, and the mutated nucleotide is located at a splicing site between the fifth intron and the sixth exon [48].

The *Arabidopsis* genome encompasses two isoforms of the catalytic subunit (POLE1A and POLE1B, also called AtPOL2A and AtPOL2B) [37,74]. The *AtPOL2A* gene, also known as *TIL1/ABO4/ESD7* [37,39,52], encodes a protein of 2161 amino acids, with a predicted molecular mass of 261 kDa. The AtPOL2B protein sequence is 79% identical (84% similar) to AtPOL2A. [37,52,74]. Both *Arabidopsis* AtPOL2 proteins possess each of the motifs necessary for a functional DNA Pol ε catalytic subunit. Only *AtPOL2A* is an essential gene [74], and it is expressed at detectable levels; loss of function of *AtPOL2B* does not affect plant growth or development, suggesting that AtPOL2A is the main active isoform during DNA replication [74]. Nevertheless, analysis of double mutants revealed that AtPOL2B is partially redundant with AtPOL2A [37,52,74]. To date, four hypomorphic alleles for *AtPOL2A* were isolated (Figure 1A): *tilted 1-4* (*til1-4*) [37], *abscisic acid oversensitive* (*abo*) *4-1* and *2* [39], and *early in short days 7* (*esd7*) [52], three of which harbor point mutations close to the catalytic site, whereas the fourth one (*abo4-2*) is a transfer DNA (T-DNA) insertion line that accumulates several truncated transcript in which one or two exons are spliced out, likely leading to the accumulation of an incomplete protein [50]. Partial loss of Pol ε results in prolonged cell cycle and S-phase [37,50], likely due to hampered fork progression. All *AtPOL2A* hypomorphic alleles display similar developmental alterations including early flowering (see below), reduced stature, and disorganized meristems, with the exception of *til1-4* in which vegetative growth after germination is less compromised than in *abo4* or *esd7* mutants, and flowering is only slightly delayed, probably because the activity of the protein is less severely compromised in this mutant [37].

Loss-of-function mutants for the *DPB2* gene arrest growth early during embryo development [37,74]. Using an over-expression strategy, we showed that excess DPB2 accumulation impairs DNA replication and causes endogenous DNA stress [49], corroborating its involvement in DNA replication. Since DPB2 is known to mediate the interaction of Pol ε with the GINS [75], our finding that altering the stoichiometry of DPB2 and POL2 affects DNA replication suggests that this role is conserved in plants. This hypothesis is corroborated by the observation of spontaneous formation of double-strand breaks (DSBs) in DPB2 over-expression (DPB2^OE^) lines that could result from fork collapse due to replisome destabilization [49]. Additional functions and regulation levels of DPB2 in other eukaryotes are reviewed in Reference [76], but whether they are conserved in plants remains to be tested. Interestingly, in *Arabidopsis*, an interaction between the CDT1 protein (a subunit of the pre-replication complex involved in the initiation of DNA replication [28]) and DPB2 was found [77]; this result is consistent with the role of Pol ε in the initiation of replication. However, this interaction was never described in other eukaryotes and its role remains unclear.

According to phylogenetic analyses, two putative homologues of DPB3, DPB3-1 (NF-YC10, nuclear factor-Y10) and DPB3-2 (NF-YC13), and one homologue of DPB4 (NF-YB11) were identified. The three proteins are part of the NF-Y family, which are sequence-specific transcription factors harboring a histone fold [78]. To date, there is no experimental data supporting the role of any of these factors as subunits of DNA Pol ε, and the plant DPB3-1 protein appears to participate in the transcriptional regulation of heat-stress genes [79,80]. How this latter function relates to DNA replication is unclear, suggesting (i) that the DPB3-1 is a bona fide NF-Y transcription factor rather than a subunit of a replicative polymerase or (ii) that it is a bifunctional protein that can participate in the two processes, like the human DPB4 subunit that is also part of the CHRAC (chromatin accessibility complex) [81]. Because the plant DPB3 and four subunits are not yet clearly identified, their function in DNA replication remains to be studied.

### 4.2. DNA Polymerase α

In all eukaryotes analyzed to date, the Pol α–primase complex is formed by four subunits, all of which are essential for cell survival. The largest subunit (POLA1/POL1) contains the DNA polymerase activity and the POLA2/POL12 subunit has no known enzymatic activity, but performs a regulatory role, likely linking the Pol α holoenzyme to components of the replication fork [82]. The other two smallest subunits harbor the DNA primase activity (PRIM1 and PRIM2, also known as PRI1 and PRI2, respectively) [83].

In plants, the first studies performed to characterize this enzyme were focused on its purification and in vitro activity. DNA Pol α was purified from several plants such as maize, wheat, pea, and cauliflower (reviewed by Bryant et al. [36]). The catalytic function of plant Pol α was demonstrated in vitro, revealing that it is capable of initiating the synthesis on single-stranded templates and of extending primers on primed templates [36]. Later, the genome sequence analysis of *Arabidopsis* and rice allowed the identification of the four putative subunits of the Pol α complex. The plant Pol α sequence is conserved compared to its yeast and animal homologues [84]. In *Arabidopsis*, *POLA1* (also known as *INCURVATA2/ICU2*) encodes the catalytic subunit, and its inactivation leads to zygotic lethality [38]. To date, only two hypomorphic alleles for the POLA1 subunit were isolated: *incurvata 2-1* (*icu2-1*) and *polα* (Figure 1B [38,41,85]), and whether the genes encoding the three other subunits are essential remains to be investigated.

### 4.3. Polymerase δ

Pol δ complex is a heterotetramer in fission yeast and animals (POLD1–4), but only three subunits were identified in budding yeast (POLD1–3) [86]. The *Arabidopsis* genome encompasses four POLD subunits (POLD1 to D4), whereas rice has two *POLD4* genes [84]. POLD1 is the catalytic subunit with polymerase and exonuclease activity, while the other subunits are involved in complex stabilization and interaction with PCNA. Pol δ also contains an associated 3′–5′ exonuclease activity, which confers a proofreading ability, and is highly stimulated by PCNA [28]. Plant Pol δ subunit expression in proliferating tissues was reported in rice and maize [87,88]. In *Arabidopsis*, as in other eukaryotes, the deletion of *POLD1* and *POLD2* genes is lethal [40,46,48]. So far, only one hypomorphic allele for *Arabidopsis* POLD1 was isolated [40] and it harbors an amino-acid substitution in the polymerase domain (Figure 1C). This *pold1* mutant also known as *gis5* (*gigantea suppressor 5*) is a thermosensitive mutant, which displays early flowering and curly leaves when grown at 24 °C and is unable to complete development at 28 °C, while these plants are identical to the wild type at 18 °C [40].

Recently, a mutation in *POLD2* (*pold2-1* mutant) was isolated [48]. This mutation changes a G to an A at a splicing site between the fifth intron and the sixth exon (Figure 1D). Consequently, *pold2-1* generates several forms of transcripts. Among them, four transcripts produce premature stop codons, and one transcript misses 6 bp, which might result in the translation of a protein with altered function. Like the *pold1* mutant, *pold2-1* plants display early flowering and are much smaller than the wild type [48].

The above-described data indicate that the subunit composition of replicative polymerases is conserved in plants like in all organisms. In addition to the developmental defects caused by impaired cell proliferation, one shared feature of the isolated mutants partially deficient for replicative polymerases is enhanced homologous recombination (HR) in somatic tissues, and upregulation of genes involved in DNA repair [39,40,41,46]. These cellular responses could be mere consequences of impaired DNA replication. However, in other eukaryotes, Pol ε and α play a direct role in replicative stress signaling (see below for details). The isolation of *Arabidopsis* hypomorphic mutants for these polymerases allowed testing the conservation of this function in planta.

## 5. Role of Plant Replicative Pols in Replicative Stress Signaling

In yeast, Pol ε (and more specifically the Pol2 subunit) is involved in the activation of the S-phase checkpoint upon replication defects such as replication fork stalling, collapse, or DNA damage [12]. Indeed, genetic analysis of various yeast mutants revealed that only the C-terminus of Pol2 that harbors no catalytic activity is essential to cell viability, notably because it mediates DNA damage response (DDR) signaling [12,89]. Pol ε is, thus, a key component of the DDR when progression of the replication fork is hampered. Pol α is also involved in the DDR [90,91]; some *pri1* mutants are unable to slow-down S-phase progression in response to DNA damaging agents [13], and later studies revealed that Pol α interacts genetically and physically with the DDR signaling machinery [91,92]. Recently, a more refined model emerged involving the activity of all three replicative polymerases in replicative stress response activation, according to which the whole replication machinery would be used to synthesize additional RNA/DNA primers and that the initial accumulation and elongation of these primers at a stalled fork would trigger checkpoint activation [93].

The DDR is highly conserved between eukaryotes with some plant-specific variations that are not detailed here as they were reviewed recently [94]. Activation of the replicative stress response relies on the ATR kinase (ATM (ataxia telangiectasia mutated) and Rad3-related, also called “Mec1” in yeast, [95]) that initiates a signaling cascade leading to cell-cycle arrest and DNA repair [94,96]. In yeast, ATR/Mec1 activation is mediated via two independent pathways, one triggered by single-stranded DNA (ssDNA) accumulation and the other requiring the C-terminal domain of Pol2a [12,97]. In plants, major contributors to the DDR acting downstream of ATR are the SOG1 (Suppressor Of Gamma 1) transcription factor, which is a master regulator of DNA repair and cell-cycle genes, and the WEE1 kinase, which inhibits cyclin-dependent kinases and stops cell-cycle progression [94].

The direct involvement of plant Pol α in DDR signaling was not investigated to date. However, we showed that the plant DNA Pol ε plays a role in replicative stress sensing upstream of ATR, as observed in budding yeast [49,50]. Indeed, the viability of *abo4* mutants and, to some extent, of DPB2^OE^ mutants depends on the components of the DNA damage checkpoint ATR and WEE1 [50]. In addition, *abo4/esd7* plants are highly sensitive to MMS (methyl-methane sulfonate, an alkylating agent) [39] and zeocin (a DNA intercalating agent causing DNA breaks), but insensitive to HU (hydroxy-urea, an inhibitor of nucleotide synthesis that causes fork stalling) [50]. These results indicate that three of the *AtPOL2A* hypomorphic alleles (*abo4-1, abo4-2*, and *esd7-1*) and excess accumulation of DPB2 trigger constitutive checkpoint activation by endogenous replicative stress, possibly by gumming up replication. Nevertheless, it is worth noting that the *til1-4* mutant also shows a prolonged cell cycle during embryo development [37], but displays contrasting features in terms of sensitivity to genotoxic agents, since it is hypersensitive to HU [50], indicating that the replicative stress response is not constitutively active in this mutant. This atypical behavior compared to all other *pol2A* mutants may be due to the fact that the mutation is within the endonuclease domain [37] and may, thus, affect the protein function differently.

**Figure 2:**
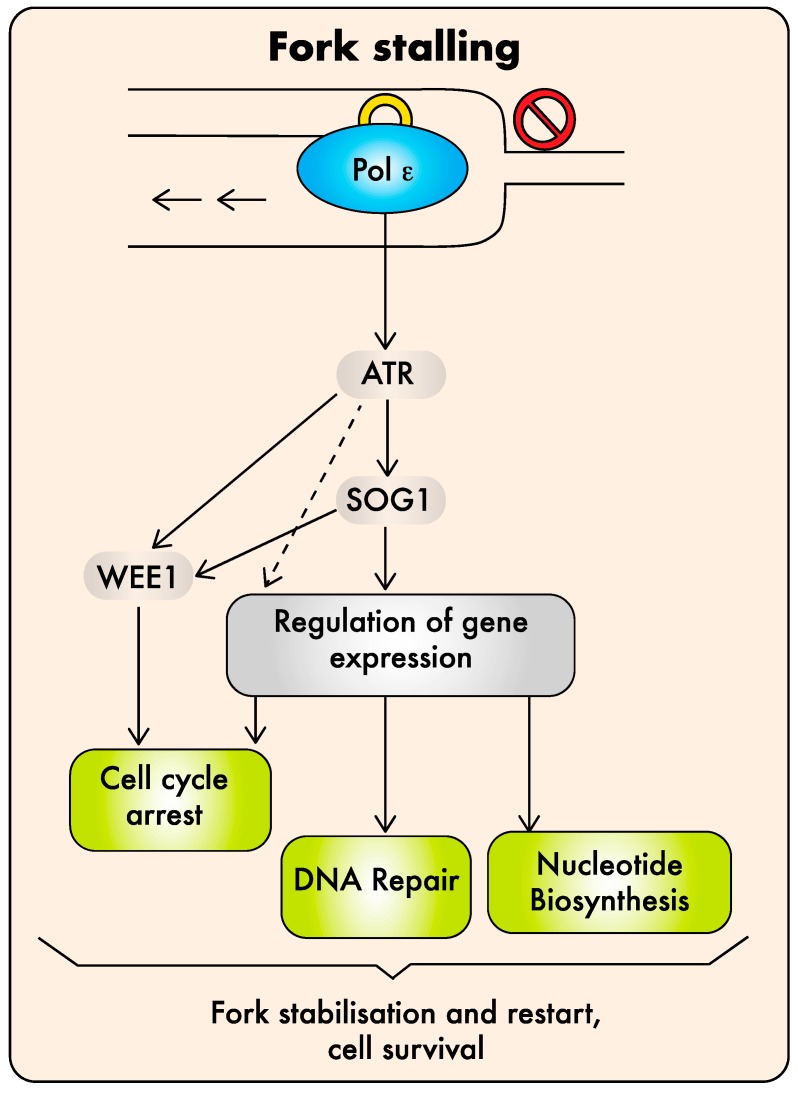
Model for Pol ε function in plant DNA damage repair (DDR). Pol ε may be directly involved in replicative stress sensing upstream of ATR to trigger checkpoint activation via the two SOG1-dependent and independent pathways, allowing the expression of genes involved in cell-cycle arrest, DNA repair, and nucleotide biosynthesis. In parallel, WEE1 contributes to arrest the cell cycle. The activation of all these mechanisms ultimately leads to fork stabilization and completion of DNA replication and cell survival [49,50].

Enhanced HR or activation of the DDR, as well as synthetic lethality with mutations affecting the replicative stress response, are expected consequences of DNA replication defects and are, therefore, not sufficient to conclude that Pol ε plays a direct role in DDR signaling. Final confirmation of the direct role of AtPOL2A in replicative stress sensing came from the observation that *AtPOL2A* knock-down plants do not display constitutive activation of the replicative stress checkpoint but, on the contrary, are hypersensitive to HU [50]. Detailed genetic analysis revealed that the DNA Pol ε-dependent pathway involves ATR, SOG1, and WEE1 to activate the replicative stress response; ATR and WEE1, but not SOG1 or ATM (the DDR kinase involved in DSB sensing), are required for the viability of the *abo4* mutants, and their tolerance to HU is at least partly mediated by SOG1 [50]. Altogether, these results indicate that the plant Pol ε may be directly involved in replicative stress sensing upstream of ATR, triggering checkpoint activation via the two SOG1-dependent and independent pathways previously described, leading to the induction of cell-cycle arrest and DNA repair [96,98]. The model for Pol ε contribution to the plant DDR is shown in Figure 2. How it functions at the molecular level in plants remains to be established. In yeast, the sensor role of DNA Pol ε likely involves its ability to interact with the checkpoint protein Rad17 [99] and the mediator protein Mrc1/Claspin [100]. However, this mechanism may differ in plants since Rad17 [101], but not claspin, seems to be conserved in plant genomes.

## 6. Putative Roles of Replicative Polymerases in Somatic and Meiotic DNA Repair

In all eukaryotes, Pol ε and Pol δ also participate in different DNA repair mechanisms that require long patches of DNA synthesis, such as base excision repair (BER), nucleotide excision repair (NER), and DSB repair (reviewed in References [81,102]). In plants, the involvement of plant Pol ε and δ in DNA repair remains to be directly investigated. Nevertheless, both Pol ε and Pol δ hypomorphic mutants are hypersensitive to DSB-inducing agents [48,50]. In addition, the *AtPOL2A* gene is upregulated by ATM in response to gamma irradiation [103], and was recently identified as a direct target of SOG1 [44]. Interestingly, *DPB2* over-expression does not compromise DSB repair but, on the contrary, enhances tolerance to DSB inducing agents such as zeocin [49]. This finding would imply that the stoichiometry of the Pol ε complex [104] is less crucial for DNA repair than for DNA replication, possibly because it does not require interaction with the full replisome. Consistently, in yeast, Dpb2 is not required for Pol2A catalytic activity in vitro, although it improves its stability [105] and enhances the fidelity of DNA replication [70].

Similarly, in rice, *POLD1* is upregulated in response to ultraviolet (UV) treatment, indicating a function in response to DNA damage in somatic cells [87]. *POLD4* was also found to be upregulated in response to bleomycin or gamma-irradiation [103,106], and only the *POLD4* gene is a direct target of SOG1. Interestingly, in human, two forms of Pol δ exist, Pol δ3 and Pol δ4; Pol δ3 is more abundant during S-phase due to specific degradation of p12 (the human POLD4), and Pol δ4 seems to be involved in HR (reviewed in Reference [107]). This control of Pol δ activity could allow regulating HR during DNA replication to avoid illegitimate recombination events. Whether the same mechanism operates in plant cells remains to be tested, but the observation that *POLD4* is a target upregulated in response to DSBs in an SOG1-dependent manner argues for a conservation of the role of Pol δ during HR.

All plant replicative polymerases were also reported to play an important role during meiosis, providing further evidence for their probable contribution to DSB repair; *AtPOL2A* mutation *(abo4-2*) and meiosis-specific POL2A RNAi, as well as DPB2 over-expression, led to an extensive chromosomal fragmentation during meiosis [50,51], and a similar phenotype was reported in hypomorphic Pol δ and Pol α mutants [42,47]. In most of these cases, the meiotic DNA fragmentation was shown to be largely dependent on SPO11-1 (SPORULATION11), which is the enzyme responsible for the formation of DSBs required for the HR process driving chromosome pairing during meiotic prophase I [42,47,51]. The authors, therefore, concluded that these polymerases are required for the repair of programmed DSBs. Although this finding is not surprising in the case of Pol δ and ε, it is unexpected for Pol α as it is not required for HR, and its involvement in DSB repair is still debated [108]. How it contributes to meiotic DSB repair in plants will, thus, require further investigation. It is also worth mentioning that the *spo11* mutation only partially rescues meiotic defects in hypomorphic DNA pol mutants and DPB2^OE^ lines [42,49,50]. In addition, the *sog1* mutation partially rescued the fragmentation phenotype of both DPB2^OE^ and *abo4-2* mutants [49,50], suggesting that the DNA fragmentation results at least partly from the SOG1-dependent activation of programmed cell death (PCD), rather than of failure to repair SPO11-dependent breaks. Interestingly, similar SPO11-independent meiotic defects were reported in various mutants deficient for replisome subunits such as CDC45 RNAi lines and *rpa* mutants [31,34], and POLE4/DPB4 subunit deficiency in mice [73] leads to p53-dependent embryonic lethality, suggesting that activation of PCD in response to defects in DNA replication is a conserved feature in all eukaryotes.

Finally, plant Pol α contributes to the stability of the genome by ensuring telomere maintenance. In *Arabidopsis*, an impaired function of Pol α leads to shorter and more heterogeneous telomeres, impacting their structure and maintenance [44]. In yeast, during telomere replication, telomerase and Polα are recruited to the chromosome termini through the (CTC1–STN1–TEN1) complex. Telomerase can catalyze the addition of telomeric repeats at the 3′ end of a telomeric sequence, and Pol α can start the synthesis of the opposite strand to generate new complete telomeres (reviewed in Reference [109]). Consistently in *Arabidopsis*, disruption of *STN1* leads to telomeric defects similar to the ones observed in *icu2-1*, supporting the notion that STN1 and Pol α may act in the same telomere maintenance process in plant cells. Interestingly, the *stn1 pol α* double mutant displays more severe developmental defect and genome instability than each single mutant, suggesting that STN1 and Pol α can also function separately in plant [44].

Overall, the data summarized above highlight the conserved role of replicative DNA Pols in the maintenance of genome integrity. Interestingly, there is accumulating evidence that they also play a crucial role in the replication of chromatin marks and, thus, in the maintenance of the epigenome.

## 7. Role of Plant Replicative Polymerases in the Maintenance of the Epigenetic Information

One striking observation about plant DNA polymerases is that most hypomorphic mutants deficient for these enzymes were identified in genetic screens that were completely unrelated to DNA replication. Indeed, the *esd7* and *pold1* (also known as *gis5* for suppressor of *gigantea* 5) mutants were isolated while searching for regulators of flowering time [40,52], while *polα* and *pold2-*1 were isolated in a genetic screen aimed at identifying components of the transcriptional gene silencing (TGS) machinery [41,48], and *abo4* mutants were initially characterized for their increased sensitivity to abscisic acid (ABA) [39]. Detailed analysis revealed that ABA sensitivity and defects in the control of flowering time are also observed in Pol α mutants [43,45], and release of TGS was reported in Pol ε mutants [39,110] (Table 2).

**Figure 3:**
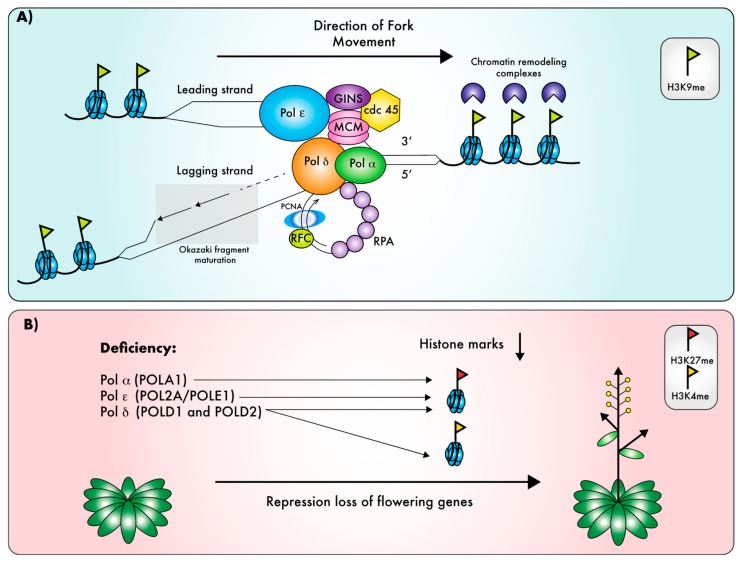
Roles of replicative DNA polymerases in the maintenance of epigenetic information. In addition to replicating DNA, the three plant replicative DNA Pols are involved in the replication of chromatin marks. (**A**) During DNA replication, chromatin is disrupted ahead of the replication fork, and the epigenetic information must be restored behind the fork, in order for chromatin marks to be inherited through DNA replication. (**B**) Hypomorphic mutants for replicative DNA polymerases showing early flowering caused by de-repression of flowering genes, due to defects in the maintenance of the inhibitory histone marks H3K27me for mutants of three polymerases [38,39,40,41,43,48,52,53]. In addition, loss of Pol *δ* mutants also affects the active H3K4me mark [40].

One possible explanation for these seemingly unrelated phenotypic defects could be that they reflect the role of replicative Pols in the replication of chromatin marks. Indeed, during DNA replication, chromatin is disrupted ahead of the replication fork, and the epigenetic information must be restored behind the fork (Figure 3A), in order for chromatin marks to be inherited through DNA replication [111,112]. Detailed genetic analysis of the *abo4-1* and *esd7-1* mutants revealed that their early flowering phenotype is due to changes in the expression of key flowering genes, as a consequence of defects on the deposition of the repressive histone mark H3K27me3 [39,52]. Likewise, Polα deficiency results in an early flowering phenotype [38,41] that was shown to originate from loss of H3K27me3-dependent repression of several flowering-specific genes [38,43]. This mark is deposited by polycomb repressing complex (PRC) proteins [113]. Interestingly, *AtPOL2A* interacts genetically with genes encoding proteins involved in chromatin dynamics such as *LHP1* (*LIKE-HETEROCHROMATINPROTEIN1*), encoding a component of the PRC, and *FAS2*. The latter encodes a subunit of the CAF-1 (chromatin assembly factor) complex, a histone chaperone involved in chromatin packaging and DNA replication. The *fas2* mutation suppresses the *esd7-1* early flowering phenotype, whereas the *lhp1* mutation aggravates it [52]. The epistatic relationship established between *FAS2* and *AtPOL2A* indicates that, in the absence of a functional CAF-1 complex, gene de-repression could not take place in *esd7-1*, suggesting that the CAF-1 complex acts downstream of AtPOL2A by facilitating the assembly of nucleosomes on newly replicated DNA [52]. More recently AtPOL2A was reported to interact both genetically and physically with PRC2 components such as CURLY LEAF (CFL), the catalytic subunit, EMF (EMBRYONIC FLOWER), and MSI1 (MULTICOPY SUPRESSOR OF IRA1). A domain of the C-terminal region of AtPOL2A mediates the binding to the different PRC2 components, and this interaction is necessary for the proper recruitment of PRC2 to flowering gene loci such as *FT* and *SOC1*, thereby regulating flowering time through the maintenance of the H3K27me3 mark on these genes (Figure 3B [53]). This observation is consistent with the recent finding that polycomb-dependent gene silencing is maintained through replication-coupled histone modification [114]. *POLA1/ICU2* also genetically interacts with *LHP1*, *CLF*, and *FAS1* [38], and deficiency in POLA1 results in reduced LHP1 binding at some of its target loci [43]. Nevertheless, direct interaction in vivo between LHP1 and Pol α catalytic subunit was not detected [43], and it, thus, remains to be determined whether plant Pol α directly associates with chromatin modifiers like Pol ε, or whether the reported defects in the maintenance of epigenetic marks are an indirect effect of disturbed DNA replication. In fission yeast, the DNA Polα catalytic subunit physically interacts with proteins involved in genes silencing, and loss of this interaction results in the de-repression of heterochromatin loci [115,116,117], suggesting that yeast Pol α directly contributes to the inheritance of chromatin marks, and that such a mechanism could be conserved in plants.

In addition to the detailed analysis of the impact of replicative Pol deficiency on H3K27me3 deposition at loci controlling flowering time, there is some evidence that other chromatin marks are also impacted in hypomorphic pol mutants; H3K4me3 levels are modified at flowering loci in Pol δ mutants [40], transposable elements are re-activated in all replicative Pol mutants [39,41,48,110], and *POLA1* regulates TGS through the deposition of H3K9me2 [41]. Furthermore, the *Arabidopsis* POL2A protein interacts with various chromatin modifiers aside of PRC components [53]. In fission yeast, Pol2 interacts with the CLCR complex [118] that mediates H3K9 methylation [119], and this interaction is crucial for assembly of heterochromatin during S-phase; disruption of the Pol ε complex results in severe loss of H3K9me and heterochromatin silencing [118,120]. Very recently, the yeast Pol ε complex and, more specifically, its accessory subunits DPB3 and 4 were shown to provide a platform for the recruitment of chromatin modifiers and remodelers including the CLCR complex during DNA replication, which in turn ensures the accurate inheritance of heterochromatin marks [120]; plant DPB3 and DPB4 may, thus, function in a similar way. Unlike Pol α and Pol ε, a role of Pol δ in the replication of chromatin marks was not reported in other eukaryotes and could, thus, be unique to plants. However, a dysfunctional Pol δ may disturb the entire replication machinery including recruitment of chromatin modifiers, which may in turn influence the reproduction of chromatin states. In line with this hypothesis, a number of mutants deficient for replisome subunits are deficient for TGS [121,122,123]. Further studies will be needed to dissect the molecular mechanisms via which components of the DNA replication machinery participate in this process.

Although Yin and colleagues reported that exogenous ABA application induced DNA damage accumulation in *abo4* mutants and not in the wild type, suggesting that ABA hypersensitivity could also result from DNA damage accumulation [39], it is tempting to speculate that the relationship between Pol ε and α and PRC-dependent gene silencing may also account for the ABA sensitivity phenotype observed in *abo4* and *icu2-1* mutants. Indeed, the *lhp1* mutant was recently found to be hypersensitive to ABA [124], suggesting that mis-regulation of ABA-responsive genes could be the primary cause of the ABA sensitivity of Pol ε- or α-deficient lines. Interestingly, microarray analysis of *icu2-1* revealed that genes involved in salicylic acid (SA) biosynthesis and accumulation were also mis-regulated in this mutant [45], again reminiscent of defects recently described in the *lhp1* mutant [124].

Why Pol ε or α deficiency specifically affects flowering time or stress responses is unclear. However, only a few loci and chromatin marks were studied in these mutants. It is, therefore, possible that other loci or chromatin marks are affected but were overlooked because they do not result in obvious phenotypic modifications. We are, thus, missing a more global view of how replicative Pol deficiency impacts the epigenome landscape in plants.

## 8. Future Directions for Replicative Polymerase Research

Overall, recent data regarding the roles of replicative polymerases highlight several common features found in mutants deficient for either of them. These include reduced growth, increased HR that likely reflects HR-mediated rescue of blocked forks, activation of the DDR and synthetic lethality with DDR components involved in the replicative stress response, and defects in the maintenance of chromatin marks. The above-listed phenotypes can be explained either by the negative effect of the mutations on DNA replication per se, or by a more direct role of the analyzed proteins in the altered processes. To date, there is some evidence for the direct contribution of plant Pol ε to DNA damage sensing and chromatin mark replication [50,53], but the underlying molecular mechanisms remain largely elusive. The main challenge in the years to come will, thus, be (i) to dissect the respective role of each protein at the molecular level to understand which replicative polymerases directly contribute to replicative stress signaling and how they perform this function since several components playing this role in other eukaryotes appear to be missing from plant genomes, and (ii) to improve our understanding of the mechanisms connecting the reproduction of histone marks to DNA replication. Indeed, only the reproduction of the repressive mark H3K27me3 was really associated with DNA replication [114], but the H3K27me1 mark was also proposed to be reproduced during DNA replication because the histone methyl-transferases that deposit this mark bind to PCNA [125,126]. In yeast, it is clear that some marks are reproduced concomitantly with fork progression while others are re-established later during the cell cycle [127]. Dissecting how this happens in plant cells and how DNA polymerases contribute to the process will clearly be a key objective in the years to come, especially to improve our understanding of stress memory in plants [128].

It is worth noting that the depletion or deficiency of DDR components or chromatin remodeling machinery was found to be lethal in other eukaryotes but does not necessarily interfere with the viability of plants, possibly due to their amazing developmental plasticity. Indeed, they can regenerate damaged tissues through the reactivation of cell division in neighboring cells [129]. Such a mechanism could allow them to cope with a defective DDR through the replacement of cells that are damaged. This feature of plants, combined with the availability of various hypomorphic mutants, allowed in-depth genetic analysis that would be difficult to perform in other models. For instance, genetic interactions between replicative polymerases were reported; *pol2a polα* plants do not have additive effects on plant growth, suggesting that both work in the same pathway [48,52]. By contrast, *pold2-1 polα* plants were smaller and exhibited more severe growth phenotypes than single mutants, suggesting that the two polymerases have additive effects on plant growth and development. Finally, the *pold2-1 pol2a* double mutant is phenotypically similar to *pold2-1*, indicating that POLD2 has an epistasis effect on Pol ε for controlling plant development [48]. However, a synergistic effect was observed during meiotic recombination in a *POLD1 RNAi pol2a* double mutant, suggesting that these polymerases have different roles in this process [47]. These double mutants could be investigated in more detail to unravel the shared and unique roles between the three DNA polymerases, whose study in other eukaryotes is limited.

## 9. Non-Replicative DNA Polymerases, Shared and Unique Functions

In all eukaryotes, non-replicative polymerases are more numerous than the replicative ones. They fulfil two main functions: (i) they contribute to DNA repair independently of DNA replication, and (ii) they allow DNA replication to proceed pass DNA lesions through a process called translesion synthesis.

## 10. Role of Non-Replicative Polymerases in TLS

A huge diversity of DNA lesions has the potential to stop fork progression. These impediments to DNA replication can be overcome in different ways. Firstly, DNA replication can be re-initiated beyond the lesion, a mechanism that is particularly frequent on the lagging strand since its synthesis is already discontinuous. PRIMPOL seems to play a critical role in this process in human cells [18]. One PRIMPOL homologue was recently identified in *Arabidopsis*, but it remains to be functionally characterized [66].

Alternatively, replacement of the replicative polymerase by a TLS polymerase with a looser catalytic site can allow the fork to progress through the lesion; this process frequently involves two TLS polymerases, one allowing the synthesis of DNA opposite the lesion, and the other performing the elongation of the DNA strand before the replisome switches back to the replicative polymerase [18]. In human, all non-replicative polymerases are involved in TLS, with Pol κ and ζ being specialized in the extension step of the TLS, whereas the others perform the TLS reaction per se, with their diversity allowing the cell to deal efficiently with a wide variety of DNA lesions [1]. Because their ability to accommodate modified nucleotides in their catalytic site is the intrinsic propriety that allows them to perform TLS, most of these polymerases are error-prone [1].

Compared to human, plant genomes encompass fewer putative TLS polymerases (seven vs. 13 in human, Table 2), but have at least one member of each DNA polymerase family. Because DNA-damaging agents all generate fork-blocking lesions, it can be difficult to determine whether a DNA polymerase is required for TLS, DNA repair, or both. However, a number of reports provide evidence for the involvement of several different DNA Pols in TLS. One of the most common DNA lesions occurring in cells is the oxidized base 7,8-oxoguanine (8-oxo-G) that is generated by reactive oxygen species. Like in human, the *Arabidopsis* Pol λ was demonstrated to efficiently incorporate a C opposite (8-oxo-G) in vitro [32]. Pol λ interacts with the PCNA2 protein that enhances its fidelity and efficiency, further confirming the probable role of Pol λ during replication [32].

Other relatively common lesions are bulky adducts induced by UV. In *Arabidopsis*, *rev3* mutants that are deficient for the catalytic subunit of the Pol ζ are hypersensitive to UV exposure but show no defect in the elimination of UV-induced lesions; by contrast, they show reduced BrdU incorporation into DNA after UV exposure, suggesting that plant Pol ζ is a TLS polymerase [56]. Likewise mutants lacking the REV1 polymerase or the accessory subunit of Pol ζ (REV7) were found to by hypersensitive to various DNA-damaging agents [55]. Consistently, simultaneous inactivation of Pol ζ and RAD5, which is involved in lesion skipping through template switching, resulted in extreme sensitivity of plants to genotoxic stress [58]. Furthermore, UV treatment severely inhibits cell division and induces PCD in root meristems of both Pol η and ζ mutants, providing further evidence for their significant contribution to TLS [130]. The respective roles of these TLS polymerases were confirmed by the observation that *rev7* and *rev1* mutants show a reduced mutation rate after UV exposure, whereas the mutation rate increases in Pol η-deficient mutants, indicating that Pol ζ and REV1 are involved in an error-prone bypass mechanism, whereas Pol η is involved in an error-free pathway [54]. In addition, REV1 is a direct target of SOG1, providing further evidence for its role in tolerance to DNA damage [131]. Pol η and Pol ζ tightly cooperate for TLS and may usually be enough to complete DNA replication. TLS by Pol η is activated as a first alternative to bypass the lesion; its function does not depend on DDR kinases. By contrast, ATR appears to promote TLS by facilitating recruitment of Pol ζ and may indirectly promote damage tolerance [59].

Plant genomes also encode homologues of Pol θ that is involved in both TLS and an alternative non-homologous end-joining (NHEJ) pathway of DSB repair (see below) [1]. Unlike other mutants lacking TLS polymerases that develop normally, *tebichi* mutants that are deficient for Pol θ display severe developmental defects such as reduced growth, and altered leaf shape and meristem function, likely due to a gap 2 (G2) arrest of the cell cycle [64]. Further genetic analysis of the Pol θ function revealed that the G2 cell-cycle arrest observed in *teb* mutant is dependent on ATR, and that inactivation of homologous recombination aggravates the developmental defects of *teb* mutants [63]. These findings led to the conclusion that Pol θ is likely required for normal S-phase progression and accounts for most of the TLS in the absence of externally applied stresses.

Thus, like other eukaryotes, plants possess a wide repertoire of TLS polymerases that are likely recruited to specific types of lesions, although this was not systematically investigated. Surprisingly, much less is known regarding the role of the non-replicative polymerases in DNA repair.

## 11. Role of Non-Replicative Polymerases in DNA Repair

In plants, like in all other eukaryotes, a large diversity of DNA repair mechanisms exists, most of which involve the activity of one or several DNA pols, depending on the type of lesion that needs to be repaired. These mechanisms were reviewed elsewhere [132] and are, therefore, be only briefly summarized. Lesions affecting a single nucleotide such as apuric/apyrimidic (AP) sites, oxidized or deaminated nucleotides, single-strand breaks, etc. are repaired through base excision repair (BER), whereas bulky lesions induced by UV can be repaired through direct reversal or NER (a process that also contributes to DNA demethylation) [132]. Both BER and NER leave a gap in the DNA that is ultimately filled by a DNA polymerase. In the case of BER, this gap can be filled by the incorporation of a single nucleotide (single-nucleotide or short-patch repair, SP) or a few nucleotides (long-patch repair, LP) [132]. In human, LP repair is performed by the replicative polymerases Pol δ and ε, whereas Pol β is the main gap-filling polymerase for the SP pathway of BER [1], but no homologue of this enzyme was found in the *Arabidopsis* or other plant genomes. This could suggest that Pol δ and ε are the only plant polymerases involved in BER. However, biochemical investigation of the BER pathway using total extracts of plant cells indicated that the gap-filling activity is insensitive to the replicative polymerase inhibitor aphidicolin, but sensitive to the Pol β-like polymerases inhibitor 2′,3′-dideoxycytidine 5′-triphosphate (ddCTP) [133]. This suggests that at least one non-replicative polymerase is involved in BER in plants, but it remains to be identified. At the end of the NER process, gap-filling can be performed in human either by Pol δ, Pol ε, or Pol κ [132]. One homologue of Pol κ was described in *Arabidopsis* [62], and the corresponding gene is a direct target of SOG1 [131,134], but it is not characterized functionally.

In addition to modified nucleotides that accumulate notably due to oxidative stress (e.g., 8-oxo-G) or UV exposure (e.g., CPD), cells also have to deal with mismatches that can arise through replication errors or deamination of methylated cytosines [132]. The mismatch repair pathway, MMR, involves a sophisticated machinery that recognizes the mismatch, discriminates between the parental and daughter strand, and excises the nucleotides on the daughter strand [132]. DNA resynthesis is thought to be performed by Pol δ, although bioinformatics analyses using the STRING database [135] predict that Pol ε interacts with the MMR machinery [136].

Another type of lesion is the formation of intra- or inter-strand crosslink. These lesions can be skipped by the replisome through re-initiation downstream of the lesion and repaired after DNA replication [18]. Pol ζ is clearly involved in the repair of all these types of lesions as it interacts genetically with several components of the repair machinery such as the MUS81 endonuclease that is involved in the excision of the lesion [57].

Finally, plant cells also have to deal with DSBs that are considered as particularly dangerous lesions since they can result in complete loss of genetic information. They can be repaired through HR (especially when an undamaged template is available in the G2 phase of the cell cycle), non-homologous end joining (NHEJ, which can be divided into classical and alternative NHEJ), and single-strand annealing (SSA) [132]. Again, in plants, it is not clear how the labor is distributed between replicative and non-replicative polymerases. As mentioned above, replicative polymerases all appear to play a role in the repair of meiotic DBSs. In addition, mutants deficient for DNA Pol λ are hypersensitive to DSB-inducing agents such as γ-irradiation or bleomycin [60]. Expression of Pol λ is high in meristems and meiotic cells, and it is induced by a variety of stress conditions [137,138,139,140]. The protein physically interacts with NHEJ components [61], further confirming its role in DSB repair. Consistently, these mutants show reduced efficiency of T-DNA integration, a process that is also considered as using the cellular machinery involved in DSB repair [60]. However, Pol θ was recently shown to be the main DNA polymerase involved in T-DNA integration through alternative NHEJ, a DSB repair mechanism in which minimal homology on a short sequence allows Pol θ to capture the single-stranded T-DNA and use it as a template to repair a pre-existing DSB in the genome [65]; strikingly, T-DNA integration is completely abolished in *tebichi* mutant, at variance with other mutants lacking DNA repair polymerases in which frequency of integration was only reduced [60]. Pol θ is also an important player of DSB repair in the absence of T-DNA as, in moss, its expression is induced by bleomycin-induced DNA damage [141].

## 12. Organellar DNA Polymerases Are Involved both in DNA Replication and Repair

Due to their endosymbiotic origin, plastids and mitochondria have their own genome, which is replicated by a dedicated machinery. The history of our understanding of organelle genome replication is very interesting and was reviewed recently [142]. Based on what is described in bacteria, and because of the evolutionary origin of organelles, organelle genomes were initially assumed to be circular, and to be replicated through a rolling circle mechanism. This model is now abandoned, because circular DNA is rare in organelles, and most of the organellar genome is actually found in the form of linear and highly complex branched molecules [142]. In this context, organellar DNA replication was proposed to be initiated through at least three mechanisms that likely co-exist: (i) recruitment of the replisome through origin binding protein and subsequent double-helix unwinding, (ii) transcription-dependent replication in which replisome recruitment is permitted by transcription-associated DNA unwinding, and (iii) a recombination-dependent process initiated by single-strand annealing [142].

Whatever their relative importance, all these initiation pathways lead to the recruitment of the organellar replisome. Like the nuclear replisome, it comprises the helicase, primase, and polymerase activities, as well as single-strand DNA-binding proteins and proteins involved in the release of mechanical constraints called gyrases. The mechanisms for organelle DNA replication were reviewed recently [142,143], and we, therefore, only briefly describe them here. The organellar replisome is relatively simple compared to its nuclear counterpart; the helicase and primase activities are likely brought by a single polypeptide called TWINKLE that has a dual targeting to plastids and mitochondria [144]. Likewise, photosynthetic eukaryotes possess one or two organellar DNA polymerases, all of which are dually targeted to plastids and mitochondria [143]. Indeed, with the exception of a few repair-related proteins, most enzymes involved in DNA metabolism are shared between plastids and mitochondria [142]. The evolutionary origin of plant organelle DNA polymerases is debated, since they appear to share more sequence identify with the *E. coli* polymerase I than with the human Pol γ protein involved in mitochondrial DNA replication; the authors, therefore, proposed to call them POPs (plant organelle DNA polymerases) [143]. *Arabidopsis*, like all other angiosperms analyzed to date, has two POP proteins called POL1A and POL1B or POPs [143]. Mutants lacking either protein are viable, but display a reduction in the organellar DNA content, whereas double mutants are lethal, suggesting that POL1A and POL1B function redundantly to allow organellar DNA replication [67]. By contrast, although the maize genome also encompasses two dually targeted POPs, loss of only one of them is sufficient to essentially abolish chloroplast DNA replication, while mitochondria genome copy number is only mildly affected, indicating that the POPs may have specialized differently in various plant species [145]. In *Arabidopsis*, only *pol1b* mutants show hypersensitivity to the gyrase inhibitor ciprofloxacin that induces DSBs in organelles, as well as genetic interaction with the ssDNA-binding proteins WHIRLY that are involved in DSB repair, indicating that it plays a more prominent role than POL1A in organelle DNA repair [67]. This model is consistent with the more recent finding that the two proteins differ in terms of fidelity, with POL1A showing an almost 10 times lower error rate compared to POL1B [68]. Both POL1A and POL1B interact with TWINKLE in the yeast two-hybrid system, and domains required for the interaction, as well as conserved amino acids likely involved in this interaction, were mapped [66], further confirming that both polymerases can insert into the replisome. Surprisingly, the same study revealed that *twinckle* null mutants are viable and show no alteration of genome copy number [66]. This result indicates that another primase can function in organelles. The authors listed a number of potential candidates including a newly identified homologue of the human PRIMPOL protein that is involved in the maintenance of both nuclear and mitochondrial genome integrity [1]. However, *primpol* single mutants are also unaffected for organellar DNA replication, and further genetic analysis will be required to determine if these two proteins function redundantly or whether other factors are involved.

Finally, contrarily to what is described for the nucleus where polymerase switching is required for DNA replication to proceed past various DNA lesions, the organelle POPs are responsible for both normal DNA replication and lesion bypass; both *Arabidopsis* POPs can efficiently replicate DNA past apurinic/apyrimidic sites [69]. Thus, the mechanisms involved in the maintenance of the organellar genome integrity involve a much more limited number of proteins than those protecting nuclear genome integrity. Interestingly, the organellar genome copy number inside one cell can vary drastically, as well as the integrity of these DNA molecules; for example, genome copy number increases dramatically early in differentiating leaf cells, whereas it decreases in older leaves, while the amount of damaged molecules increases, illustrating that the requirement for replication and repair capacity of organellar DNA varies over time, as well as between cell types and tissues [142].

## 13. Concluding Remarks

Altogether, recent findings on plant DNA polymerases demonstrate the conservation of their function compared to what is known in mammals or yeast. One limitation of the available data so far is the paucity of biochemical studies that preclude the clear identification of each polymerase’s activity. Indeed, most of our knowledge stems from the genetic analysis of mutants and tests of their sensitivity to various genotoxins, all of which directly or indirectly induce several types of DNA lesions. These kinds of approaches do not allow discriminating between TLS or DNA repair activities, or pinpointing the specific type of damage handled by a given polymerase. Future work would, thus, require developing more biochemical assays to obtain a full picture of each polymerase’s function.

Another promising direction for future research will be to consider the replication and repair process of chromatin as a whole instead of focusing only on DNA. Indeed, recent findings provide evidence for the role of the replication machinery in the maintenance of chromatin marks, but deeper analyses are needed to dissect the molecular mechanisms involved, and more specifically to determine which DNA replication complexes play a direct role in the replication of chromatin states. Likewise, DNA damage signaling and DNA repair both modify chromatin locally, and how the epigenetic status of the locus is restored after DNA repair is still largely unknown. This issue is emerging in the animal field, but receives little attention in plants, opening new research avenues for the years to come.

## Figures and Tables

**Table 1:** Nomenclature for replicative DNA polymerases (Pols) in human, yeast (*Saccharomyces cerevisiae* and *S. pombe*), and plant (*Arabidopsis thaliana*) orthologues. Catalytic subunits are indicated in bold characters.

DNA Pol	Human (Gene/Protein)	*S. Cerevisiae/S. Pombe* (Gene/Gene)	*A. Thaliana*
Pol α/primase	***POLA1*/p180**	***POL1/pol1***	***POLA1*** *(ICU2, AT5G67100)*
	*POLA2*/p70	*POL12/pol12*	*POLA2 (AT1G67630)*
	***PRIM1*/p49**	***PRI1/pri1***	***PRIM1*** *(AT1G67320)*
	*PRIM2*/p58	*PRI2/pri2*	*PRIM2 (AT5G41880)*
Pol δ holoenzyme	***POLD1*/p125**	***POL3/pol3***	***POLD1*** *(AT5G63960)*
	*POLD2*/p50	*POL31/cdc1*	*POLD2 (AT2G42120)*
	*POLD3*/p68	*POL32/cdc27*	*POLD3 (AT1G78650)*
	*POLD4*/p12	- */cdm1*	*POLD4 (AT1G09815)*
Pol ε holoenzyme	***POLE1*/p261** *POLE2*/p59 *POLE3*/p17 *POLE4*/p12	***POL2/cdc20*** *DPB2/dpb2* *DPB3/dpb3* *DPB4/dpb4*	***POL2A*** *(ABO4/ESD7, AT1G08260)* *DPB2 (AT5G22110)* *DPB3 (NF-YC13, AT5G43250;* *NF-YC10, AT1G07980)* *DPB4 (NF-YB11, AT2G27470)*

**Table 2:** Known functions of *Arabidopsis* replicative and specialized polymerases. HR—homologous recombination; DSB—double-strand break; TLS—translesion synthesis; UV—ultraviolet; NHEJ—non-homologous end-joining.

Polymerase	Cellular Function	References
B-family (Pol α, δ, ε, and ζ in human)		
Pol alpha (α)	Deficiency induces HR	[41]
	DSB repair in meiosis	[42]
	Maintenance of histone marks	[38,41,43]
	Maintenance of telomeres	[44]
	Response to abscisic acid (ABA)	[45]
Pol delta (δ)	Deficiency induces HR	[40,46]
	DSB repair in meiosis	[47]
	Maintenance of histone marks	[40,48]
	Response to DNA-damaging agents	[48]
Pol epsilon (ε)	Checkpoint signaling	[49,50]
	DSB repair in meiosis and meiotic checkpoint	[50,51]
	Deficiency induces HR	[39]
	Maintenance of histone marks	[39,52,53]
	Response to abscisic acid (ABA)	[39]
Pol zeta (ζ) *REV3* (AT1G67500) *REV7* (AT1G16590)	TLS (UV-induced lesions) Repair or intra and inter-strand crosslink	[54,55,56,57,58,59] [57]
X-family (Pol λ, β, μ, and TdT in human)		
Pol lambda (λ)	TLS (8-oxo-G)	[32]
(AT1G10520)	DSB repair	[60,61]
Pol eta (η) POLH (AT5G44740)	TLS (UV-induced lesions)	[54]
Y-family (Pol κ, ι, η, and REV1 in human)		
Pol kappa (κ) (AT1G49980)		[62]
Pol Rev1 (AT5G44750)	TLS (UV-induced damage)	[54,55]
A-family (Pol θ, γ, and ν in human)		
Pol theta (θ)	TLS (required for normal progression of DNA replication)	[63,64]
POLQ (AT4G32700)	DSB repair through alternative NHEJ	[65]
Archaeo-eukaryotic primase family PrimPol		
PRIMPOL (AT5G52800)	Not functionally characterized yet, putative role in organelle DNA replication	[66]
Plant organelle polymerases (POPs)		
Pol1-like A (Pol γ1, AT3G20540)	Organellar replication and repair, potentially more specifically involved in DNA replication	[67,68,69]
Pol1-like B (Pol γ2, AT1G50840)	Organellar replication and repair, potentially more specifically involved in DNA repair	[67,68,69]
